# Current Insights in the Application of Bone Grafts for Local Antibiotic Delivery in Bone Reconstruction Surgery

**DOI:** 10.7150/jbji.38373

**Published:** 2019-10-15

**Authors:** Arne Peeters, Guy Putzeys, Lieven Thorrez

**Affiliations:** 1 Faculty of Medicine, KU Leuven, Leuven, Belgium; 2 Orthopedic Centre, AZ Groeninge, Kortrijk, Belgium; 3 Department of Development and Regeneration, KU Leuven, Kortrijk, Belgium

**Keywords:** bone grafts, impregnation, antibiotic delivery, infection

## Abstract

**Introduction:** Bone implant related infection is still one of the biggest challenges in bone and joint surgery. Antibiotic impregnated bone grafts seem to be promising in both treatment and prevention of these infections. However, great variance in methodology predominates this field of research. This paper gives an overview of the published literature.

**Methods:** The PRISMA-flowchart was used as protocol for article selection. Medline was searched and articles were selected in accordance with predetermined exclusion criteria.

**Results:** Forty-eight articles were included in the synthesis. Topics including bone graft type, manipulations of the graft, elution profile, bacterial inhibition, osteotoxicity, incorporation, special impregnation methods, clinical use and storage were investigated.

Therapeutically, high initial levels seem appropriate for biofilm eradication. A single stage procedure in the treatment of bone implant related infection seems feasible. Prophylactically, the literature indicates a reduction of postoperative infections when using antibiotic impregnated bone grafts.

**Conclusion:** Bone grafts are a suitable carrier for local antibiotic application both therapeutically and prophylactically.

## Introduction

Infection is still one of the biggest challenges in bone and joint surgery. Osteomyelitis rates increased from 11.4 cases per 100000 person-years in the period from 1969 to 1979 to 24.4 per 100000 person-years in the period from 2000 to 2009.^1^ In prosthetic surgery, device-related infection accounts for 12.8% of revisions after hip arthroplasty and up to 20% after knee arthroplasty.^2^,^3^ It is expected that the prevalence will increase due to increasing comorbidities.^4^ With annually 800000 joint arthroplasties in the USA and the UK, and estimations of over 4 million arthroplasties per year by 2030, periprosthetic joint infection will be one of the major future complications.^5^ In musculoskeletal trauma surgery, fracture-related infections reach up to 30% in cases of open fracture. Life-changing consequences include permanent loss of function or amputation.^6^

A major problem in bone- and implant-infections is biofilm formation. Bacteria embedded in a biofilm are able to withstand high concentrations of bactericidal antibiotics, leading to treatment failure and infection recurrence. Local antibiotic dosages far above the minimal inhibitory concentration (MIC) are required to eradicate biofilm-associated infections.^7^ New pharmacodynamic parameters, such as the minimal biofilm eradications concentration (MBEC), are essential for an objective quantification of antibiotic activity.^8^ It has been shown that local concentrations required for biofilm eradication cannot be achieved by systemic antibiotics administration alone^9^ since the MBEC is not reached.^8^

When infections occur, the operative treatment typically is a two-stage procedure. The first stage includes removal of implants and radical debridement. Dead space management is performed by temporary filling with non-biodegradable cement spacers loaded with antibiotics. In a second stage procedure, the cement spacer is removed and new implants are inserted to repair skeletal continuity. 10,11 However, bone cements lose their function of antibiotic elution largely in the first days of implantation.^12^ In combination with their avascular nature, they are vulnerable to bacterial colonization and re-infection of the surgical site.^13^ Other disadvantages are the need for follow-up surgery to remove the non-biodegradable substance and a limited choice of antibiotics because of the heat produced during stiffening.^14^

An optimal solution for the above mentioned problems would be to locally deliver an antibiotic through a biodegradable substance with a suitable elution profile. This would raise local antibiotic levels, making it possible to eradicate biofilms without systemic adverse effects and perhaps to treat bone implant related infection in a one-stage procedure.^15^ A technique that could meet these criteria is bone grafting. While bone grafting is already widespread in reconstructive orthopedic surgery, the use in bone deficiency after infection has been seen as a contraindication since the avascular grafts are prone to re-infection.^16^ It has been suggested that antibiotic-loaded bone grafts have the appropriate characteristics for prophylactic purposes as an adjunct to systemic profylaxis.^17^

While it is theoretically clear that antibiotic-loaded bone grafts can have their value in orthopedic surgery, the clinical use is still not conclusive. Variance in methodology such as type of grafts, antibiotics, impregnation method and dose, makes it difficult to draw conclusions. This paper gives an overview of the published literature on therapeutic and prophylactic use of antibiotic impregnated bone grafts.

## Methods

The PRISMA-flowchart was used as the protocol for Medline article selection. The last search data were included in January 2019. Search terms were: "Bone Transplantation"[Mesh] AND (Local antibacterial agents OR Vancomycin OR Gentamicin OR Tobramycin), Antibiotic impregnated bone grafting, Bone cell toxicity AND Antibiotics AND Local delivery, Osteoblast AND Local antibiotics, Cancellous bone AND Vancomycin, Lyophilized bone AND Antibiotics, Iontophoresis AND Bone AND Antibiotics.

The following exclusion criteria (from title or abstract) were used: papers in which only PMMA or calcium phosphate cements were used, papers discussing tuberculosis or using demineralized bone matrix, papers older than 1990, review papers, case reports, expert opinions, papers from which the full text could not be retrieved or written in another language than English. On the other hand, clinical, animal and *in vitro* studies were included. A first screening was based on the title and abstract. A second screening for eligibility was done by full-text assessment.

## Results

**Figure 1:**
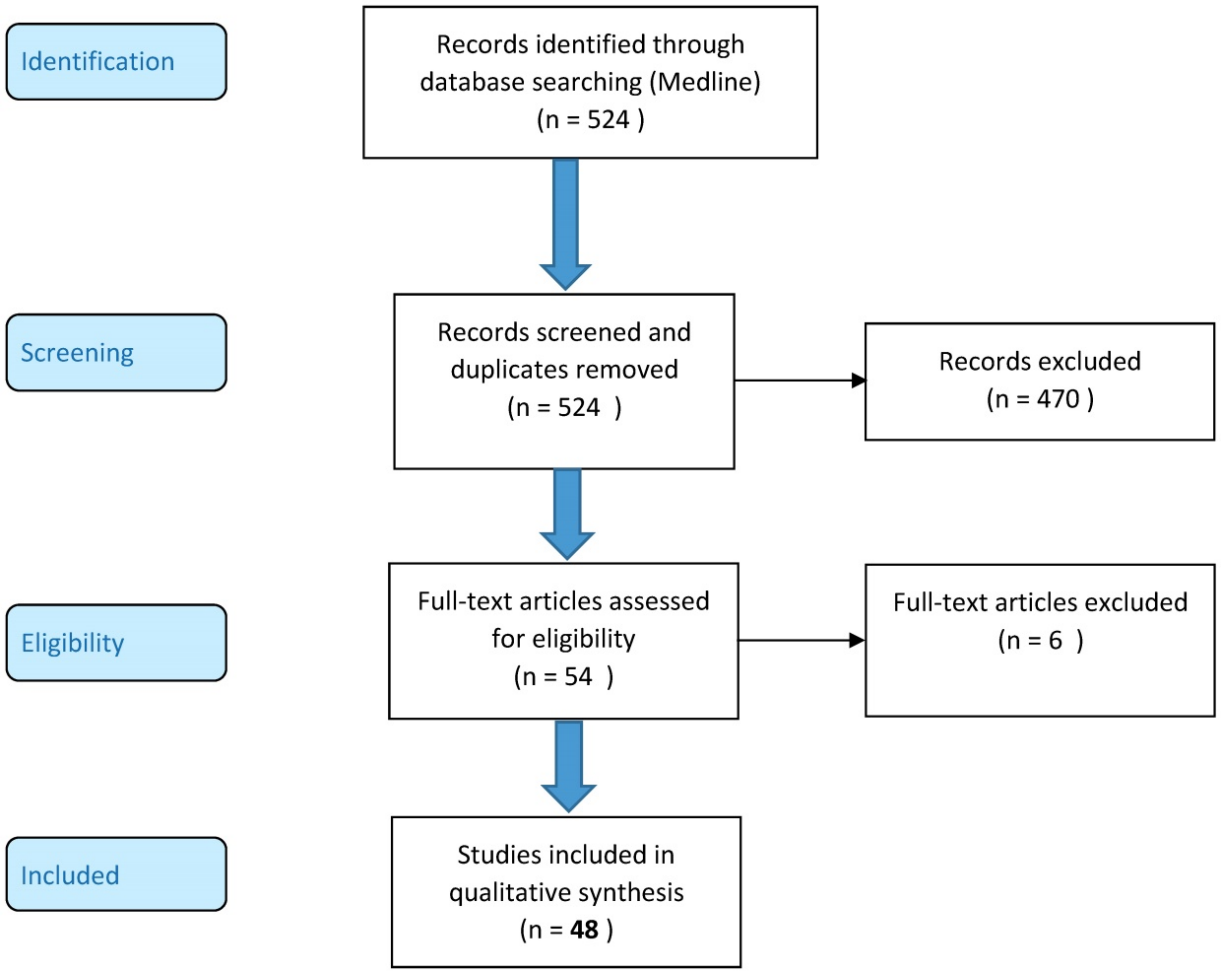
PRISMA 2009 flow diagram of the selection process.

Based on the search criteria, a total of 524 articles were identified. After the first screening, 54 articles met the selection criteria. In a second full-text screening another 6 articles were excluded. Eventually, a total of 48 articles were included in this review (*Fig 1*).

40 of the 48 articles were experiments with bone graft. These are listed in *Table S1*. 10 of the 48 articles were *in vitro* studies on cell cultures, studying the bone toxicity of antibiotics. These are listed in *Table S2*. Two studies covered both bone grafts as well as toxicity and were thus included in both tables. Based on these articles, we review following parameters: bone type, manipulations, impregnation methods, elution kinetics, osteotoxicity, incorporation, storage and current clinical evidence.

### Bone type

The suitability for impregnation of cancellous versus cortical bone grafts is defined by uptake capacity and elution profile. Most studies (28 of 40, *Table S1*) use cancellous bone grafts. Six studies using cancellous bone as antibiotic carrier explicitly conclude an adequate *in vitro* elution, suggesting cancellous bone as a suitable antibiotic carrier.^18^-^23^ Only 4 studies describe the use of cortical bone alone for impregnation.^24^-^27^ Cortical bone was impregnated with 4 different antibiotics and demonstrated an elution profile with high release in the first 24h. Netilmicin, vancomycin and rifampicin-impregnated grafts proved to be effective by completely eradicating *Staphylococcus aureus-*induced intramedullary infections of rats.^27^

A direct comparison of cancellous and cortical bone grafts was performed with vancomycin and tobramycin impregnation.^28^ After vancomycin impregnation, initial elution concentrations were significantly lower for cortical bone than for cancellous bone. At day 9, the difference in concentrations between the 2 graft types was no longer significant. Overall, the total vancomycin release was significantly lower for cortical bone. For tobramycin, cortical grafts had an initial release half of cancellous bone grafts. Concentrations went below the minimal inhibitory concentration (MIC) at day 22 for cortical grafts while cancellous bone grafts were still well above the MIC at day 28.^28^

Most studies (32 of 40, *Table S1*) used allografts. A direct comparison between allo- and autografts was not performed anywhere. Elution of vancomycin from cancellous human allografts versus bovine xenografts was similar, whereas tobramycin elution was slightly (not tested statistically) higher from bovine cancellous bone.^28^ Furthermore, the origin of the bone grafts is variable with the femoral head being the most frequent (14 of 40, *Table S1*). Half of the studies using cancellous allografts derive from femoral heads, whereas cancellous autografts are more often taken from the iliac crest. Cortical grafts originate from the femoral or tibial diaphysis.

Bone particle size varies within as well as between studies, ranging from 0.01 to 6 mm. The influence of bone fragment size on antibiotic elution was examined.^29^ Fine and coarse morselized cancellous bone were impregnated with netilmicin or vancomycin and elution was evaluated for 14 days. For netilmicin, elution was higher from fine particles but for vancomycin no significant difference was found.^29^

### Manipulation

Fresh frozen grafts are the most studied (21 of 40 studies, *Table S1*). Further manipulations can be performed to improve storage characteristics and/or reduce immunogenicity. Ten of the 48 studies discuss bone manipulation. In 6 studies, grafts were submitted to a freeze-drying process. In one study, both lyophilized and fresh frozen bone grafts were mixed with gentamicin powder.^30^ Fresh frozen bone showed a release decreasing from an initial 10000 μg/ml to 300 μg/ml at day 4. Lyophilized grafts showed a release rate from 4000 μg/ml to 400 μg/ml from 1^st^ to 3^rd^ day. These concentrations exceeded the MIC of *S. aureus*.^30^ Similar elution kinetics of gentamicin and vancomycin were found in another study comparing freeze-dried and fresh frozen bone grafts.^31^

A double lyophilization step in which a graft was freeze-dried, impregnated with vancomycin and freeze-dried again did not show a significant effect on elution.^28^ Freeze-drying the graft while still being submerged in the antibiotic solution did not prolong the elution, but the overall amount of released antibiotic was higher.^32^

Other bone graft manipulations performed for decellularization and/or sterilization included chemical cleaning processes with detergents^21^ or solvents^28^,^33^,^34^, pulse lavage with normal saline^35^,^36^, sonication, irradiation and combinations thereof. Gentamicin impregnation was not significantly affected by bone graft cleaning by washing versus detergent treatment and sonication.^37^

### Impregnation methods

The majority of papers use an antibiotic solution or dry antibiotic powder mixing as impregnation method. However, other impregnation methods have been described. Seven of 48 articles discuss non-conventional impregnation methods. Iontophoresis is a technique applying an electric potential which accelerates diffusion of certain antibiotics into bone. Sections of either sheep or human tibial diaphysis were impregnated by immersion or iontophoresis with gentamicin or flucloxacillin.^24^ Compared to the specimens which had been soaked, a larger zone of inhibition was observed for those which had been treated using iontophoresis.^24^ By varying voltages, iontophoresis time and concentrations, vancomycin elution from sheep tibial diaphysis could be modulated.^25^

Coating of bone with alginate after impregnation extended the release of amoxicillin, ciprofloxacin and vancomycin, in contrast to chitosan coating which did not significantly prolong antibiotic release.^32^

The bone surface can be modified by EDTA submersion, making more amines available at the surface, addition of a linker and then tethering of vancomycin. These vancomycin-tethered allografts did not elute active antibiotic but resisted *S. aureus* colonization for 20 days, prevented biofilm formation and did not influence osteoblast colonization or viability.^26^,^38^,^39^

A vancomycin drug delivery system made of natural bee wax and glycerin was described for autologous cancellous bone graft, resulting in a long-acting (4-6 weeks) antibiotic-impregnated bone graft. This combination succeeded in eradicating an induced *S. aureus* osteomyelitis in rats with no negative effects on incorporation.^40^

### Elution kinetics

The most important feature of a bone graft as an antibiotic carrier is the elution kinetics. Both the carrier and the antibiotic influence this characteristic. The impregnated graft should release the antibiotic resulting in local concentrations exceeding the MIC (for prophylaxis) or MBEC (for treatment) for a sufficiently long time. Eleven of 48 articles discuss elution kinetics. Elution kinetics were described for cancellous fresh frozen bone grafts mixed with vancomycin powder (1% w/w). Elution was well above the MIC for *S. aureus* during 16 days, with a maximum of 499.7 μg/ml at day 2-4.^22^ In another study, highly purified cancellous grafts impregnated with vancomycin (100 mg/ml) reached initial concentrations up to 20000 μg/ml.^28^ *In vivo* use of vancomycin-impregnated grafts however was not successful in eradicating an induced *S. aureus* osteomyelitis in rats.^41^ In contradiction, bone grafts mixed with a vancomycin-enriched wax successfully eradicated an induced *S. aureus* osteomyelitis in rats.^40^

Moxifloxacin solution-impregnated fresh frozen grafts released 3846.9 μg/ml the first day and exceeded the MIC for 42 days.^20^ Tobramycin solution-impregnated cancellous bone grafts (80 mg/ml) initially released 10000 μg/ml and remained above the MIC for 28 days.^28^ Gentamicin solution-impregnated grafts were characterized by a rapid release of gentamicin during the first 2 days, exceeding 1000 μg/ml. At day 4, half of the absorbed antibiotic was eluted. Overall drug concentrations remained above the MIC for 14 days.^21^ A significant higher release of gentamicin was observed when using gentamicin-sulfate versus a gentamicin-sulfate/palmitate mixture.^42^ Release rates of antibiotics such as benzylpenicillin, dicloxacillin, cephalothin, netilmicin, vancomycin, ciprofloxacin, clindamycin and rifampicin were examined by incubating fresh morselized bone with solutions of these antibiotics.^18^ The first three (beta-lactams) showed very high values with a rapid decline within 7 days. Netilmicin, vancomycin, ciprofloxacin and clindamycin were released within 14 days. Only rifampicin remained higher than the MIC for 21 days.^18^ These results were confirmed *in vivo* by intramuscular implantation in rats. Clindamycin, netilmicin, vancomycin and rifampicin impregnated bone had a bacteriostatic effect for 7 days, ciprofloxacin for 3 days and benzylpenicillin and cephalothin only for 1 day.^19^

Variables such as antibiotic concentration of the impregnation fluid, time used for impregnation and pH of the impregnation fluid have been investigated. Time of impregnation of cancellous bone did not influence netilmicin elution but was beneficial for vancomycin and moxifloxacin, indicating a difference in absorbance kinetics.^20^,^29^ A fourfold increase in impregnation concentration caused a 252% and 125% increase in release of netilmicin or vancomycin respectively.^29^ Gentamicin impregnated at 10 versus 1 mg/ml gave significantly higher release during the first 2 days, by day 4 elution concentrations were similar.^21^ Bone impregnated at pH 3 or pH 5 eluted significantly lower amounts of antibiotics than bone impregnated at pH 7.^29^ Impregnation duration was also investigated for cortical bone.^27^ Increasing impregnation time from 1 to 10 h increased the total amount of released netilmicin, vancomycin and rifampicin 3-4 fold. Further increase to 100 h of impregnation led to a 6-10 fold increase of released antibiotic. For ciprofloxacin this influence was smaller with a 2-3 fold increase.^27^

When compared to antibiotic loaded bone cement, bone has more favorable elution kinetics. Cancellous bone released substantially higher antibiotic concentrations during the first 3 days and the duration of detectable elution was comparable. Moreover, in contrast to allogeneic bone grafts, a significant amount of antibiotics remained in the cement. 31

### Osteotoxicity

Applying antibiotics at concentrations toxic to osteoblasts may possibly undermine bone healing. Therefore, antibiotic release should not exceed the bone toxicity threshold. Ten of 48 articles (*Table S2*) investigated the effects of antibiotics on cell lines. In a comparative study, osteoblasts were incubated with solutions ranging from 0 to 5 mg/ml with one of several antibiotics.^43^ Cell number and osteogenic activities were determined. Vancomycin was found to be the least cytotoxic and other well-performing antibiotics were amikacin and tobramycin. Antibiotics causing the greatest cell toxicity were rifampin, minocycline, doxycycline, nafcillin, penicillin, ciprofloxacin, colistin methanesulfonate and gentamicin. For these antibiotics, concentrations ≥200 µg/ml already negatively influenced the cell number.^43^

Comparable findings were described for preosteoblasts and prechondrocyte cell lines.^44^ Proliferation was severely affected after 48 hours exposure to ciprofloxacin ≥100 µg/ml and to vancomycin and tobramycin ≥2000 µg/ml.^44^ This toxic dose of vancomycin was also described in another study where 10000 μg/ml vancomycin caused cell death on an osteoblastic cell line, while 1000 μg/ml had no effect.^45^ Similarly, tobramycin levels ≥400 µg/ml reduced osteoblast cell replication and 10000 µg/ml caused cell death.^46^

For cefazolin concentrations up to 100 μg/ml, little or no effect was seen on osteoblastic cell growth, but at 1000 μg/ml growth decreased.^45^ On mesenchymal stromal cells, cell reduction was noticed at doses ≥250 µg/ml for 24h or even at ≥50 µg/ml when sustained for 72h.^47^ Cefuroxime, another cephalosporin, had little effect on human osteoblast proliferation below 100 µg/ml. Concentrations of 250 and 1000 µg/ml even stimulated proliferation. Cytotoxicity was observed when the concentration of 1000 µg/ml was sustained more than 48h.^48^

For gentamicin, exposure up to 800 μg/ml for 48 h did not change preosteoblast cell number.^49^ However; longer exposure (5-10d) of concentrations ≥250 μg/ml to rat osteoblasts negatively influenced cell growth.^21^

Clindamycin increased calcification by human osteoblasts at 10 and 25 µg/ml, whereas ≥50 µg/ml decreased calcification. Proliferation decreased in a dose-dependent manner reaching 3.5% of control samples at 500 µg/ml for 72 h.^50^

Noteworthy, proliferation can also be influenced by the carrier, independent of antibiotic loading; freeze-dried bone decreased proliferation of mesenchymal stem cells as compared to fresh-frozen grafts.^31^

### Incorporation

Even more relevant than measurement of osteotoxicity *in vitro* is incorporation of bone graft into the host bone *in vivo*. Twelve of 48 articles discussed incorporation. Incorporation of tobramycin-impregnated bone grafts (impregnation concentrations up to 800 mg/ml) was evaluated in dogs.^51^ Histomorphological tests after 4 weeks of incorporation showed no significant difference between low and high drug concentrations.^51^ Comparable results were described for cancellous bone impregnated with tobramycin powder which displayed comparable incorporation to plain bone grafts as measured radiographically, microscopically and biomechanically.^52^

Also vancomycin impregnation of bone grafts did not change bone healing*.* Grafts with or without vancomycin were implanted in a pig tibial defect. No differences were found by radiography, histopathology and immunohistology between the 2 groups.^53^ In the presence of osteomyelitis, vancomycin-impregnated grafts also did not influence bone mineral content and density.^41^

Incorporation of cefazolin-impregnated cancellous bone allografts was studied in goats. After 12 weeks, no significant histomorphometrically differences between an antibiotic impregnated graft and a control graft could be found.^35^

When clinically applied in fracture surgery, the incidence of nonunion did not increase as the overall incidence of nonunion remained within normal range.^54^-^57^ Fourteen and 20 months after revision of total hip arthroplasty with vancomycin-impregnated bone grafts, bone samples were equal to those in allografts without vancomycin.^58^ In revision of infected tibial nonunions, no recurrent nonunion was observed when using antibiotic-impregnated autografts as compared to unmodified cancellous autografts where one nonunion occurred.^59^ In one stage revision of periprosthetic infection, incorporation of vancomycin and tobramycin impregnated grafts was similar to conventional grafting.^34^

### Storage

Bone chips impregnated with gentamicin, cefazoline or vancomycin and stored for 1-6 months at -20°C or -80°C all retained the same antibiotic activity as compared to freshly prepared samples.^60^,^61^

### Clinical evidence for prophylactic use

Four of 48 articles discussed prophylactic use. Vancomycin-impregnated allografts were investigated as prophylactic measure in aseptic one-stage revision of total hip arthroplasties.^62^,^63^ Patients were treated with impaction grafting using vancomycin powder-impregnated allografts and PMMA as fixation. Plain PMMA or PMMA with gentamicin or tobramycin was used. Vancomycin reached local levels of 1400 µg/ml and nephrotoxicity was not observed. Vancomycin-supplemented bone allografts reached local concentrations 20-300 times higher than the MIC for *S. aureus* until 48 hours, without impairing renal function. The presence of tobramycin or gentamicin in the cement enhanced the bactericidal effect.^62^ In a follow-up study, vancomycin-impregnated allografts and plain PMMA was evaluated on 75 patients, undergoing a revision hip arthroplasty. Incidence of infection was analyzed and compared to data of traditional impaction grafting using antibiotic loaded cement. The result was 1 post-operative infection, an incidence similar to antibiotic loaded cement.^63^

Prophylactic use of gentamicin powder-impregnated bone grafts was clinically evaluated in a retrospective study on 220 cerebral palsy patients who underwent spinal fusion surgery. Hundred fifty four children underwent fusion with gentamicin-impregnated bone grafts and the other 66 patients with antibiotics-free bone grafts. 3.9% of the gentamicin impregnated bone graft patients developed a deep wound infection, compared to 15.2% without antibiotics.^64^

Iontophoresed cortical allografts have prophylactically been used in 31 patients undergoing revision arthroplasty or limb salvage surgery. Grafts were iontophoresed with flucloxacillin and gentamicin. Postoperative antibiotic levels in the drain fluid were well above the MIC of *S. aureus* and mean serum antibiotic levels were low. There were two complications within 6 months, a wound infection and an early non-union. Late onset complications were 2 allograft infections, 3 late fractures and 3 established non-unions.^56^ A direct comparison to non-impregnated grafts has not been performed.

### Clinical evidence for therapeutic use

Eight of 48 articles discussed therapeutic use. Vancomycin-supplemented bone allografts were tested in a two-stage treatment of infected hip arthroplasty. 55 Thirty hips were treated by implant removal and parenteral antibiotic therapy. In a second stage, reconstruction was performed using vancomycin powder-impregnated bone grafts. Complications were 1 further infection, 1 periprosthetic fracture, 2 single dislocations and 4 displacements of the greater trochanter. At the latest follow-up, 29 hips showed radiographic evidence of consolidation.^55^ In a similar study, 18 tibial nonunions were treated in a two stage procedure.^57^ The first stage consisted of debridement, filling with gentamicin PMMA bead chains and stabilization with external fixation. During the second stage, antibiotic beads were removed and reconstruction was performed using vancomycin powder-impregnated cancellous autografts. Infection control was obtained in all patients. Nonunion occurred in 5 patients, where an additional procedure was needed but eventually all patients showed good consolidation of the grafted bone.^57^

Clinical evaluation of vancomycin-impregnated bone grafts was taken one step further by investigating the possibility of a one-stage revision of infected implants.^33^,^34^ Forty eight revision procedures were executed by a one-stage debridement, lavage and filling with lyophilized antibiotic-impregnated cancellous allografts. No cement was used. The procedures included revision of infected total hip replacement, total knee replacement or intramedullary nailing. Antibiotics used for impregnation were vancomycin or tobramycin, depending on the micro-organism. Post-operative vancomycin serum levels were 0 - 4.2 μg/ml, drain levels 8 - 2243 μg/ml. Renal function showed no differences. Recurrence of infection was observed in 2 cases. There was no loosening or dislocation of implants. 33,34,58

A direct comparison of antibiotic powder-impregnated autografts with unmodified cancellous autografts was performed on 96 patients with an infected tibial nonunion*.*^59^ All patients underwent a first procedure of debridement and filling with antibiotic-impregnated PMMA bead chains. In a second procedure antibiotic bead chains were removed and the defect was reconstructed. Either antibiotic-impregnated cancellous autografts or plain cancellous autografts were used. The chosen antibiotic was specific to the responsible pathogen, mostly vancomycin and piperacillin. Follow-up for 4 to 6 years indicated that bone union was achieved in all patients that received impregnated autografts. Only two infections developed in this group, resulting in an infection arrest rate of 95.6%. In the group receiving plain autografts the infection arrest rate was significantly worse at 82%.^59^

Use of iontophoresed grafts in septic conditions in a study of 12 two-stage revisions in which the second stage was performed with flucloxacillin and gentamicin iontophoresed bone grafts resulted in no further infections.^54^

One clinical study describes the use of antibiotic-impregnated bone grafts for maxillofacial surgery. Peri-implantitis was treated in a one-stage procedure of debridement immediately followed by filling with vancomycin and tobramycin-impregnated allografts. After 12 months the bone defect was significantly reduced, no continuous bone loss could be observed and none of the implants had to be replaced.^65^

## Discussion

The large variation in methodology makes it difficult to compare outcomes systematically. Cancellous bone is the preferred bone graft type in small bone defects^66^ and also for antibiotic impregnation it seems superior. Cortical bone is less accessible to antibiotics than cancellous bone and resulted in lower antibiotic elution concentrations.^28^ Allografts are most commonly used, but from the perspective of antibiotic binding and elution, both human and bovine bone perform similar.^28^ As a result, only general principles can guide towards the best choice. Xenografts require processing to eliminate potential disease transmission and enhance biocompatibility. Processing can also be applied to the more commonly used allografts. Autograft bone is without risk of immunogenicity but its use is restricted by its limited quantity and donor site morbidity.^51^,^66^

Bone graft treatment changes the characteristics: lyophilization results in more open space due to elimination of blood and fat. A lyophilized bone graft can be conserved long-term at room temperature and is less antigenic.^67^ No significant difference was found in elution profile of gentamycin and vancomycin between impregnated morselized bone and freeze-dried bone powder.^31^ This is in contrast to the claim that bone marrow removal and freeze drying dramatically increases antibiotic uptake capability.^28^

Special impregnation methods may be useful in modifying the antibiotic elution profile. Iontophoresis can be used to impregnate grafts more thoroughly, reaching higher elution concentrations.^24^ To prolong release, an antibiotic-enriched bone wax or polysaccharide coating can be used.^32^,^40^ Antibiotic-tethered bone grafts could be useful in circumstances of high infection risk. The bonded antibiotic could for example ensure a prolonged protection of the avascular grafts.^39^

Vancomycin was found to be the least osteotoxic antibiotic.^43^,^44^ It has a favorable elution profile both *in vitro* and *in vivo,* which may be related to its high molecular weight.^18^,^19^,^68^ Vancomycin does not influence the rate of bone incorporation.^41^,^53^,^58^ Clinically, there was a low incidence of postoperative infection in both septic and aseptic interventions.^55^,^63^ Vancomycin levels were high locally but remained low systemically.^33^,^34^ However, vancomycin should be used cautiously; it is used for infections caused by organisms resistant to penicillins or cephalosporins and unnecessary usage may lead to increased rates of resistance.^69^ Additionally, vancomycin has a strict gram-positive spectrum.^70^ To obtain a broad-spectrum coverage for general use, an additional antibiotic with gram-negative efficiency such as gentamicin or tobramycin should be added. Gentamicin has a suitable elution profile and significantly lowered infections in patients.^42^,^64^ On the other hand tobramycin appeared to be less osteotoxic compared to gentamicin,^43^ did not affect bone incorporation^51^,^52^ and combination with vancomycin is clinically promising.^33^,^65^

Although locally high antibiotic concentrations can be established initially, a concern is potential toxicity. Comparison of initial elution concentrations to results of *in vitro* osteotoxicity studies indicates that initial antibiotic concentrations can reach levels toxic to osteoblasts.^21^,^28^,^44^ However, *in vivo* the rate of bone incorporation was never affected 51,53 and clinical studies demonstrated no increase in nonunion.^34^,^58^,^59^ Systemically, low serum levels were determined 34,56 and adverse effects such as ototoxicity, hepatotoxicity and nephrotoxicity were not observed.^34^,^62^

Currently, only two related impregnated bone products are commercially available.^71^ These are allogenic cancellous bone grafts cleaned and processed using supercritical CO_2_ and impregnated with respectively vancomycin or tobramycin. Lyophilization is performed to optimize storage life.^72^ After rehydration, initial local concentrations are very high, with up to 20000 µg/ml for vancomycin and up to 13000 µg/ml for tobramycin.^28^ These high antibiotic levels seem appropriate for elimination of biofilm, making it possible to cure bone infection in a one-stage procedure.^33^,^34^,^65^ Noticeable is that other studies do not reach these high local concentrations. 20-22 It is stated that mainly the highly purified character of the grafts contributes to these high concentrations.^73^

Important to note is that many studies still compare to the MIC to declare appropriate antibacterial inhibition. This is likely relevant for conditions where no prior infection was present, however for biofilm eradication higher concentrations are necessary.^74^ Biofilms display specific biological properties compared to bacteria in planktonic state. Biofilm communities produce an extracellular matrix which reduces both effectiveness of host defensive mechanisms as well as penetration of antibiotics.^26^ Several authors indicate that biofilm formation can be prevented by using antibiotic impregnated bone.^21^,^26^,^38^ Moreover, one-stage revision with antibiotic impregnated bone seems feasible. 33,34,59,65 Comparing the elution kinetics to the MBEC would be more relevant *in situ*ations where biofilm-related infections need to be treated.

As suggested by Frommelt *et* al., bone grafts can be suitable for prophylactic use.^75^ The elution profile is characterized by high levels during the first days, preventing contamination. Later levels drop but are still multiple weeks above the MIC. This creates a toxic bactericidal environment for the remaining sensitive organisms.^20^-^22^ The first clinical studies are promising, clearly lowering the rate of infections.^56^,^62^-^64^

## Conclusion

Despite great variance in methodology, we conclude that bone grafts have been found suitable for local antibiotic application. Several approaches resulted in high initial antibiotic concentrations, essential for prophylaxis and even biofilm eradication. Although high antibiotic concentrations induce osteoblast toxicity *in vitro*, no evidence was found that impregnation of bone with antibiotics would induce osteoblast toxicity and affect bone incorporation *in vivo*. Applied prophylactically, antibiotic-impregnated bone grafts were associated with low infection rates in complex cases and therapeutically, a single stage procedure for the treatment of bone infection appears to have some benefit but more studies are needed. Notwithstanding these initial promising results, studies comparing several protocols remain sparse and independent validation of results in larger cohorts would be warranted.

## Supplementary Material

Supplementary figures and tables.
