# Supplementary material for: Current Insights in the Application of Bone Grafts for Local Antibiotic Delivery in Bone Reconstruction Surgery
Source: J Bone Jt Infect. 2019 Oct 15;4(5):245–53. doi: 10.7150/jbji.38373 (PMC6831806; doi:10.7150/jbji.38373)
Supplement: Supplementary file 1 — Supplementary figures and tables. [file jbjiv04p0245s1.pdf]

Table S1. Summary of the studies describing a bone graft experiment.

Ref.	Author	Title	Journal	Year	In vitro/ In vivo/ clinical	Allograft/ Autograft/ Xenograft	Cortical/ cortico-cancellous/ cancellous	Manipulation	Origin of bone graft	Particle size	Antibiotic(s)	Impregnation method	Quantity of antibiotic	concentration	impregnation time
51	Barckman J.	Bone allograft and implant fixation tested under influence of bio-burden reduction, periosteal augmentation and topical antibiotics. Animal experimental studies.	Dan Med J	2014	In vivo	Allograft	/	Fresh frozen	Proximal humerus, proximal tibia, distal femur of 2 dogs	1 mL	Tobramycin	Solution	0.25 ml	200 mg/mL; 800 mg/mL	5 min
64	Borkhuu B, Borowski A, Shah SA, Littleton AG, Dabney KW, Miller F.	Antibiotic-loaded Allograft Decreases the Rate of Acute Deep Wound Infection After Spinal Fusion in Cerebral Palsy.	Spine	2008	Clinical	Allograft	Cortico-cancellous	Lyophiliz-ation	/	30-60 mL	Gentamicin	Solution	/	8–10 mg/kg patient	/
55	Buttaro MA, Pusso R, Piccaluga F.	Vancomycin-supplemented impacted bone allografts in infected hip arthroplasty. Two-stage revision results.	J Bone Joint Surg Br	2005	Clinical	Allograft	Cancellous	Fresh frozen	Human femoral heads	0.4-0.6 cm	Vancomycin	Powder	/	500 mg/fe-moral head	15 min
58	Buttaro MA, Morandi A, Rivello HG, Piccaluga F.	Histology of vancomycin-supplemented impacted bone allografts in revision total hip arthroplasty.	J Bone Joint Surg Br	2005	Clinical	Allograft	Cancellous	Fresh frozen	3 human femoral heads	0.4-0.6 cm	Vancomycin	Powder	/	1000 mg/fe-moral head	15 min
62	Buttaro MA, Gimenez MI, Greco G, Barcan L, Piccaluga F.	High active local levels of vancomycin without nephrotoxicity released from impacted bone allografts in 20 revision hip arthroplasties.	Acta Orthop	2005	Clinical	Allograft	Cancellous	Fresh frozen	56 human femoral heads	0.4-0.6 cm	Vancomycin	Powder	/	1000 mg/fe-moral head	15 min
63	Buttaro MA, Guala AJ, Comba F, Suarez F, Piccaluga F.	Incidence of deep infection in aseptic revision THA using vancomycin-impregnated impacted bone allograft.	Hip Int	2010	Clinical	Allograft	Cancellous	Fresh frozen	224 human femoral heads	0.5-1 cm	Vancomycin	Powder	/	1000 mg/fe-moral head	15 min

53	Buttaro M, González Della Valle A, Piñeiro L, Mocetti E, Morandi A, Piccaluga F.	Incorporation of vancomycin-supplemented bone allografts Radiographical, histopathological and immunohistochemical study in pigs.	Acta Orthop Scand	2003	In vivo	Allograft	Cancellous	Fresh frozen	6 g out of humeral metaphysis	0.4-0.6 mm	Vancomycin	Powder	20 mg	/	15 min
59	Chan YS, Ueng SW, Wang CJ, Lee SS, Chen CY, Shin CH.	Antibiotic-impregnated autogenic cancellous bone grafting is an effective and safe method for the management of small infected tibial defects: a comparison study.	J Trauma	2000	Clinical	Autograft	Cancellous	/	Iliac crest	/	Vancomycin Piperacillin	Powder	/	/	/
57	Chen C-E, Ko J-Y, Pan C-C.	Results of vancomycin-impregnated cancellous bone grafting for infected tibial nonunion.	Arch Orthop Trauma Surg	2005	Clinical	Autograft	Cancellous	/	5.6-39.6 g out of iliac crest	/	Vancomycin	Powder	500 mg	/	/
37	Coraça-Huber DC, Hausdorfer J, Fille M, Steidl M, Nogler M.	Effect of two cleaning processes for bone allografts on gentamicin impregnation and in vitro antibiotic release.	Cell Tissue Bank	2013	In vitro	Allograft	Cancellous	/	Human femoral heads	5-10 mm	Gentamicin	Powder	/	5 g/1 g	1h
30	Coraça-Huber DC, Ammann CG, Nogler M, Fille M, Frommelt L, Kühn K-D, et al.	Lyophilized allogeneic bone tissue as an antibiotic carrier.	Cell Tissue Bank	2016	In vitro	Allograft	Cancellous	Lyophiliz-ation, Fresh frozen	Human femoral heads	1g	Gentamicin Vancomycin	Powder	/	specified in article	1 min
42	Coraça-Huber DC, Putzer D, Fille M, Hausdorfer J, Nogler M, Kühn K-D.	Gentamicin palmitate as a new antibiotic formulation for mixing with bone tissue and local release.	Cell Tissue Bank	2014	In vitro	Allograft	Cancellous	Fresh frozen	Human femoral heads	0.01 –5 mm	Gentamicin (*GS= Gentamicin sulfate, GP= Gentamicin palmitate)	Powder	/	0,1 g GS+GP/1 g; 0,1 g GS/1g*	1 min
60	Coraça-Huber DC, Wurm A, Fille M, Hausdorfer J, Nogler M, Kühn K-D.	Effect of freezing on the release rate of gentamicin palmitate and gentamicin sulfate from bone tissue.	J Orthop Res	2014	In vitro	Allograft	Cancellous	Fresh frozen	Human femoral heads	/	Gentamicin	Frozen, Powder	/	GS 1%,3%,5 %; GS+GP 1%,3%,5 %	0; 1mo; 6mo
24	Day RE, Megson S, Wood D.	Iontophoresis as a means of delivering antibiotics into allograft bone.	J Bone Joint Surg Br	2005	In vitro	Allograft, Xenograft	Cortical	Fresh frozen	Tibial diaphysis	20 mm long	Flucloxacillin Gentamicin	Iontophoresis	/	10 mg/ml 10 mg/ml	1 min/ 5 min/ 10 min
25	Edmondson MC, Day	Vancomycin iontophoresis of	Bone	2014	In vitro	Xenograft	Cortical	Fresh	Tibial	20	Vancomycin	Iontoph-	/	Variable	Varia

	R, Wood D.	allograft bone.	Joint Res					frozen	diaphysis	mm long		oresis			
32	Hornyák I, Madácsi E, Kalugyer P, Vácz G, Horváthy DB, Szendrői M, et al.	Increased release time of antibiotics from bone allografts through a novel biodegradable coating.	Biomed Res Int	2014	In vitro	Allograft	Cancellous	Lyophiliz-ation	Human femoral heads	0.05 g	Amoxicillin Ciprofloxacin Vancomycin	Solution Chitosan/ alginate coating	1 mL	10 mg/mL	24h
20	Kanellakopoulou K, Sahinides T, Tsaganos T, Galanakis N, Giamarellou H, Giamarellos-Bourboulis EJ.	In vitro elution of moxifloxacin from cancellous bone allografts.	J Biomed Mater Res Part A	2010	In vitro	Allograft	Cancellous	Fresh frozen	Human femoral heads	1 g	Moxifloxacin	Solution	5 mL	100 mg/mL	1h/2 4h/4 8h
26	Ketonis C, Barr S, Adams CS, Hickok NJ, Parvizi J.	Bacterial colonization of bone allografts: establishment and effects of antibiotics.	Clin Orthop Relat Res	2010	In vitro	Allograft	Cortical	EDTA	/	1 mL	Vancomycin	Solution AEEA-linker	/	10 mg/mL	/
38	Ketonis C, Barr S, Adams CS, Shapiro IM, Parvizi J, Hickok NJ.	Vancomycin bonded to bone grafts prevents bacterial colonization.	Antimicrob Agents Chemother	2011	In vitro	Allograft	Cortico-cancellous	EDTA	/	0.5-2 mm	Vancomycin	Solution AEEA-linker	/	10 mg/mL	/
39	Ketonis C, Barr S, Shapiro IM, Parvizi J, Adams CS, Hickok NJ.	Antibacterial activity of bone allografts: Comparison of a new vancomycin-tethered allograft with allograft loaded with adsorbed vancomycin.	Bone	2011	In vitro	Allograft	Cortico-cancellous	EDTA	Femoral shafts	0.5-2 mm, 2 mL	Vancomycin	Solution AEEA-linker	/	10 mg/mL	12-16h
56	Khoo PPC, Michalak KA, Yates PJ, Megson SM, Day RE, Wood DJ.	Iontophoresis of antibiotics into segmental allografts.	J Bone Joint Surg Br	2006	Clinical	Allograft	Segmental	Irradiated	/	/	Gentamicin Floxacillin	Iontoph-oresis	/	10 mg/ml 40 mg/ml	20 min
31	Kucera T, Ryskova L, Soukup T, Malakova J, Cermakova E, Mericka P, et al.	Elution kinetics of vancomycin and gentamicin from carriers and their effects on mesenchymal stem cell proliferation: an in vitro study.	BMC Musculoskelet Disord	2017	In vitro	Allograft	Cancellous	Lyophiliz-ation, Fresh frozen	Proximal tibia	/	Vancomycin Gentamicin	Solution	10 mL	50 mg/mL 8 mg/mL	30 min
21	Lewis CS, Katz J, Baker MI, Supronowicz PR, Gill E, Cobb RR.	Local antibiotic delivery with bovine cancellous chips.	J Biomater Appl	2011	In vitro	Xenograft	Cancellous	Sterilized irradiated	Long bones	1-2 mm	Gentamicin	Solution	100 µL	1, 5, 10 mg/ml	/
52	Lindsey RW, Probe R, Miclau T, Alexander JW, Perren SM.	The effects of antibiotic-impregnated autogeneic cancellous bone graft on bone healing.	Clin Orthop Relat Res	1993	In vivo	Autograft	Cancellous	Fresh frozen	Femoral diaphysis	3 g	Tobramycin	Powder	90 mg	30 mg/g	/

61	Mathijssen NM, Petit PL, Pilot P, Schreurs BW, Buma P, Bloem RM.	Impregnation of bone chips with antibiotics and storage of antibiotics at different temperatures: an in vitro study.	BMC Musculos kelet Disord	2010	In vitro	Allograft	Cancellous	Fresh frozen	Human femoral heads	0.1 g	Cefazolin<br>Clindamycin<br>Linezolid<br>Oxacillin<br>Vancomycin	Frozen (-20°C, -80°C)	/	6.0 mg/ml<br>2.5 mg/ml<br>3.0 mg/ml<br>0.1 mg/ml<br>7.0 mg/ml	1mo; 6mo; 1y
35	Mathijssen NM, Hannink G, Pilot P, Schreurs BW, Bloem RM, Buma P.	Impregnation of bone chips with alendronate and cefazolin, combined with demineralized bone matrix: a bone chamber study in goats.	BMC Musculos kelet Disord	2012	In vivo	Allograft	Cancellous	Fresh frozen	Sternum of donor goats	1-2 mm	Cefazolin	Solution, Alendro-nate impregn-ation	5 mL	200 µg/mL	10 min
22	Melicherčík P, Jahoda D, Nyč O, Klapková E, Barták V, Landor I, et al.	Bone grafts as vancomycin carriers in local therapy of resistant infections.	Folia Microbiol	2012	In vitro	Allograft	Cancellous	Fresh frozen	Human femoral heads	4.5 mm	Vancomycin	Powder	/	0.1g /10g	15 min
23	Melichercik P, Klapkova E, Landor I, Judl T, Sibek M, Jahoda D.	The effect of Vancomycin degradation products in the topical treatment of osteomyelitis.	Bratisl Lek Listy	2014	In vitro	Allograft	Cancellous	Fresh frozen	Human femoral heads	4.5 mm	Vancomycin	Powder	/	0.25g/10 g	35 min
54	Michalak, KA, Khoo PPC, Yates, PJ, Day RE, Wood DJ	Iontophoresed segmental allografts in revision arthroplasty for infection.	J Bone Joint Surg Br	2006	Clinical	Allograft	Segmental	Irradiated	Femoral and tibial	/	Gentamicin<br>Floxacillin	Iontopho resis	/	10 mg/ml<br>40 mg/ml	20 min
65	Nart J, de Tapia B, Pujol À, Pascual A, Valles C.	Vancomycin and tobramycin impregnated mineralized allograft for the surgical regenerative treatment of peri-implantitis: a 1-year follow-up case series.	Clin Oral Investig	2017	Clinical	Allograft	Cancellous	Fresh frozen	/	/	Tobramycin<br>Vancomycin	/	/	/	/
41	Ozturk AM, Tabak AY, Aktekin CN, Altay M, Erdemli E, Karahuseyinoglu S, et al.	Alendronate enhances antibiotic-impregnated bone grafts in the treatment of osteomyelitis.	Int Orthop	2008	In vivo	Xenograft	/	Fresh frozen	/	/	Vancomycin	Solution, Alendro-nate impregn-ation	10 mL	100 mg/ml	10 min
36	Shah MR, Patel RR, Solanki R V, Gupta SH.	Estimation of drug absorption in antibiotic soaked bone grafts.	Indian J Orthop	2016	Clinical	Allograft	Cancellous	Fresh frozen	shavings of total knee replacement	0.5 mL	Genamicin<br>Vancomycin	Solution	/	Variable	Varia

28	Winkler H, Janata O, Berger C, Wein W, Georgopoulos A.	In vitro release of vancomycin and tobramycin from impregnated human and bovine bone grafts.	J Antimicrob Chemother	2000	In vitro	Allograft<br>Xenograft	Cancellous<br>Cortical	Lyophiliz-ation<br>Irradiated	Human femoral and proximal tibia	1-5 mm	Tobramycin<br>Vancomycin	Solution	/	80 mg/ml<br>100 mg/ml	24h
33	Winkler H, Kaudela K, Stoiber A, Menschik F.	Bone grafts impregnated with antibiotics as a tool for treating infected implants in orthopedic surgery<br>– one stage revision results.	Cell Tissue Bank	2006	Clinical	Allograft	Cancellous	Irradiated	Cadaveric donors	2-8 mm	Tobramycin<br>Vancomycin	Solution	/	75 mg/ml<br>100 mg/ml	/
34	Winkler H, Stoiber A, Kaudela K, Winter F, Menschik F.	One stage uncemented revision of infected total hip replacement using cancellous allograft bone impregnated<br>with antibiotics.	J Bone Joint Surg Br	2008	Clinical	Allograft	Cancellous	Lyophiliz-ation	Tibial and femoral metaphysis	2-8 mm	Tobramycin<br>Vancomycin	Solution	/	75 mg/ml<br>100 mg/ml	/
18	Witsø E, Persen L, Løseth K, Bergh K.	Adsorption and release of antibiotics from morselized cancellous bone. In vitro studies of 8 antibiotics.	Acta Orthop Scand	1999	In vitro	Allograft	Cancellous	/	/	15 g	Benzylpenicillin<br>Dicloxacillin<br>Cephalotin<br>Netilmicin<br>Vancomycin<br>Ciprofloxacin<br>Clindamycin<br>Rifampicin	Solution	20 mL	100 mg/ml<br>100 mg/ml<br>100 mg/ml<br>100 mg/ml<br>50 mg/ml<br>2 mg/ml<br>150 mg/ml<br>60 mg/ml	10 min
19	Witsø E, Persen L, Løseth K, Benum P, Bergh K.	Cancellous bone as an antibiotic carrier.	Acta Orthop Scand	2000	In vitro<br>In vivo	Xenograft	Cancellous	/	/	20 g	Benzylpenicillin<br>Cefalotin<br>Clindamycin<br>Netilmicin<br>Vancomycin<br>Ciprofloxacin<br>Rifampicin	Solution	20 mL	100 mg/ml<br>100 mg/ml<br>150 mg/ml<br>100 mg/ml<br>50 mg/ml<br>2 mg/ml<br>60 mg/ml	10 min
29	Witsø E, Persen L, Benum P, Bergh K.	Release of netilmicin and vancomycin from cancellous bone.	Acta Orthop Scand	2002	In vitro<br>In vivo	Allograft	Cancellous	/	/	/	Netilmicin<br>Vancomycin	Solution	10 mL	100 mg/ml<br>400 mg/ml<br>100 mg/ml	10 min

27	Witsø E, Persen L, Benum P, Bergh K.	Cortical allograft as a vehicle for antibiotic delivery.	Acta Orthop	2005	In vitro	Allograft	Cortical	/	Human femoral cortical bone	1.46 g	Netilmicin Vancomycin Ciprofloxacin Rifampicin	Solution	3 mL	400 mg/mL 100 mg/mL 2 mg/mL 50 mg/mL	1h, 10h, 100h
40	Zahar A, Kocsis G, Citak M, Puskás G, Domahidy M, Hajdú M, Antal I, Szendrői M.	Use of antibiotic-impregnated bone grafts in a rabbit osteomyelitis model.	Technol Health Care	2017	Animal	Autograft	Cancellous	/	Tibia	/	Vancomycin	Drug delivery system (dds)	/	2 mg dds /mg bone graft	/

Table S2. Summary of studies describing cell line experiments.

ref.	Author	Title	Journal	Year	in vitro/animal/clinical	Cell lines	Antibiotic(s)	Concentrations	Impregnation time	Results
44	Antoci V, Adams CS, Hickok NJ, Shapiro IM, Parvizi J.	Antibiotics for Local Delivery Systems Cause Skeletal Cell Toxicity In Vitro.	Clin Orthop Relat Res	2007	in vitro	MC3T3- E1 preosteoblasts, N1511 prechondrocytes	Ciprofloxacin Tobramycin Vancomycin	0, 25, 50, 75, 100, 150, 200, 250 µg/mL 0, 125, 250, 500, 1000, 2000, 4000 µg/mL 0, 125, 250, 500, 1000, 2000, 4000 µg/mL	48h	Osteoblast DNA toxicity: Ciprofloxacin: 25 µg/mL Tobramycin: 125 µg/mL Vancomycin: 250 µg/mL Chondrocyt DNA toxicity: Ciprofloxacin: 200 µg/mL Tobramycin: 125 µg/mL Vancomycin: 125 µg/mL
45	Edin ML, Miclau T, Lester GE, Lindsey RW, Dahners LE.	Effect of cefazolin and vancomycin on osteoblasts in vitro.	Clin Orthop Relat Res	1996	in vitro	MG-63 osteoblast	Cefazolin Vancomycin	0, 10, 100, 1000, 10000 µg/mL	24h/72h	Decreased cell growth Vancomycin: 10000 µg/mL Decreased cell growth Cefazolin 24h: 400 µg/mL Decreased cell growth Cefazolin 72h: 200 µg/mL Cell dead Cefazolin: 10000 µg/mL
49	Ince A, Schütze N, Karl N, Löhr JF, Eulert J.	Gentamicin negatively influenced osteogenic function in vitro.	Int Orthop	2007	in vitro	C2C12 cells	Gentamicin	12.5, 25, 50, 100, 200, 400, 800 µg/mL	48h	Cell viability: 25 µg/mL Cell number: not significant Alkaline phosphatase activity: 12.5 µg/mL Gene expression: not significant
31	Kucera T, Ryskova L, Soukup T, Malakova J, Cermakova E, Mericka P, et al.	Elution kinetics of vancomycin and gentamicin from carriers and their effects on mesenchymal stem cell proliferation: an in vitro study.	BMC Musculoskelet Disord	2017	in vitro	Mesenchymal Stem Cells (BM MSC) Dental Pulp Stem Cells (DPSC)	Gentamicin (in carrier) Vancomycin (in carrier)	8 mg/mL 40 mg/mL	17d	Fresh frozen: not significant Freeze-dried: negative effect on MSC proliferation
21	Lewis CS, Katz J, Baker MI, Supronowicz PR, Gill E, Cobb RR.	Local antibiotic delivery with bovine cancellous chips.	J Biomater Appl.	2011	in vitro	Rat calvarial osteoblasts	Gentamicin (in solution/ in antibiotic infused cancellous bone)	100, 250, or 500 mg/mL 5, 20, or 40 mg/g	10d	The 100mg/mL solution was the only concentration that was statistically similar to the 0 mg/mL media There were no statistically significant differences in the levels of alkaline phosphatase.

46	Miclau T, Edin ML, Lester GE, Lindsey RW, Dahners LE.	Bone toxicity of locally applied aminoglycosides.	J Orthop Trauma	1995	in vitro	MG-63 osteoblast	Tobramycin	0, 10, 100, 200, 400, 800, 1000, 10000 µg/mL	24h/72h	Decreased cell growth: 400 µg/mL<br>Cell death: 10000 µg/mL
50	Naal FD, Salzmann GM, von Knoch F, Tuebel J, Diehl P, Gradinger R, et al.	The effects of clindamycin on human osteoblasts in vitro.	Arch Orthop Trauma Surg	2008	in vitro	Human osteoblasts isolated from trabecular bone specimens	Clindamycin	0, 10, 25, 50, 100, and 500 µg/ml	24h/48h/ 72h	Decreaded proliferation after 24h: 500 µg/mL<br>Decreased proliferation after 72h: dose-dependent decrease<br>Increaded LDH activity after 48h: 500 µg/mL<br>Increased ALP activity after 24h: 10 µg/mL<br>Decreaded ALP activity after 24h: 500 µg/mL<br>Matrix calcification increase: 10 and 25 µg/mL<br>Matrix calcification decrease: 50, 100 and 500 µg/mL
47	Pilge H, Fröbel J, Lensing-Höhn S, Zilkens C, Krauspe R.	Cefazolin Irreversibly Inhibits Proliferation and Migration of Human Mesenchymal Stromal Cells.	Biomed Res Int	2016	in vitro	Bone marrow mononuclear cells	Cefazolin	0, 50, 100, 250, 500, 1000 µg/mL	24h/48h/ 72h	MSC migration decrease:<br>24h: 500 and 1000 µg/mL<br>48h and 72h: 100 to 1000 µg/mL<br>MSC proliferation decrease:<br>24h: 250 to 1000 µg/mL<br>48h and 72h: 50 to 1000 µg/mL
43	Rathbone CR, Cross JD, Brown K V., Murray CK, Wenke JC.	Effect of various concentrations of antibiotics on osteogenic cell viability and activity.	J Orthop Res	2011	in vitro	Human osteoblasts	21 different AB	0, 10, 100, 200, 500, 1000, 2000, 5000 µg/mL	10d/14d	Vancomycin, amikacin and tobramycin were the least toxic.<br><br>Rifampin, minocycline, doxycycline, nafcillin, penicillin, ciprofloxacin, colistin methanesulfonate and gentamicin were the most toxic.Concentrations ≤200 µg/ml negatively influenced the cell number.

48	Salzmann GM, Naal FD, von Knoch F, Tuebel J, Gradinger R, Imhoff AB, et al.	Effects of cefuroxime on human osteoblastsin vitro.	J Biomed Mater Res Part A	2007	in vitro	Human osteoblasts isolated from trabecular bone specimens	cefuroxime	25, 50, 100, 250, 1000 µg/mL	24h/48h/ 72h	Increase proliferation 24h/48h/72h: 250 and 1000 µg/mL<br>LDH activity 24h/48h/72h: 1000 µg/mL<br>ALP activity increase: 50 and 100 µg/mL (48h)<br>ALP activity decrease: 100 and 1000 µg/mL (24h)<br>Matrix calcification increase: x4: 25 and 50 µg/mL ; x2: 250 µg/mL<br>Matrix calcification decrease: 1000 µg/mL
